# Magnetic Core-Shell Iron Oxides-Based Nanophotocatalysts and Nanoadsorbents for Multifunctional Thin Films

**DOI:** 10.3390/membranes12050466

**Published:** 2022-04-26

**Authors:** Viorica Muşat, Nicolae Stănică, Elena Maria Anghel, Irina Atkinson, Daniela Cristina Culiţă, Silviu Poloşan, Lenuţa Crintea (Căpăţână), Alina Cantaragiu Ceoromila, Cristian-Teodor Buruiană, Oana Carp

**Affiliations:** 1 Laboratory of Chemical Nanotechnologies-LNC-CNMF, “Dunărea de Jos” University of Galati, 111 Domnească Street, 800201 Galaţi, Romania; lenuta.crintea@ugal.ro; 2 Institute of Physical Chemistry, “Ilie Murgulescu” of Romanian Academy, Spl. Independenţei 202, 060021 Bucharest, Romania; nstanica@icf.ro (N.S.); irinaatkinson@yahoo.com (I.A.); dculita@icf.ro (D.C.C.); ocarp@icf.ro (O.C.); 3 Multifunctional Materials and Structures Laboratory, National Institute of Materials Physics, Atomistilor 405 A, 077125 Magurele, Romania; silv@infim.ro; 4 Cross-border Faculty, “Dunărea de Jos” University of Galaţi, 111 Domnească Street, 800201 Galaţi, Romania; alina.cantaragiu@ugal.ro; 5 Department of Chemistry, Physics and Environment, Faculty of Sciences and Environment, “Dunărea de Jos” University of Galați, 111 Domnească Street, 800201 Galați, Romania; cristian.buruiana@ugal.ro

**Keywords:** iron oxides, core–shell, co-precipitation, sol-gel, superparamagnetic nanoparticles, photocatalysis, nano-sorption, thin film

## Abstract

In recent years, iron oxides-based nanostructured composite materials are of particular interest for the preparation of multifunctional thin films and membranes to be used in sustainable magnetic field adsorption and photocatalysis processes, intelligent coatings, and packing or bio-medical applications. In this paper, superparamagnetic iron oxide (core)-silica (shell) nanoparticles suitable for thin films and membrane functionalization were obtained by co-precipitation and ultrasonic-assisted sol-gel methods. The comparative/combined effect of the magnetic core co-precipitation temperature (80 and 95 °C) and ZnO-doping of the silica shell on the photocatalytic and nano-sorption properties of the resulted composite nanoparticles were investigated by ultraviolet-visible (UV-VIS) spectroscopy monitoring the discoloration of methylene blue (MB) solution under ultraviolet (UV) irradiation and darkness, respectively. The morphology, structure, textural, and magnetic parameters of the investigated powders were evidenced by scanning electron microscopy (SEM), X-ray diffraction (XRD), Raman spectroscopy, Brunauer–Emmett–Teller (BET) measurements, and saturation magnetization (vibrating sample magnetometry, VSM). The intraparticle diffusion model controlled the MB adsorption. The pseudo- and second-order kinetics described the MB photodegradation. When using SiO_2_-shell functionalized nanoparticles, the adsorption and photodegradation constant rates are three–four times higher than for using starting core iron oxide nanoparticles. The obtained magnetic nanoparticles (MNPs) were tested for films deposition.

## 1. Introduction

Due to magnetic, optical and electrical properties, biocompatibility, eco-friendliness and low price [1,2,3,4], iron oxide nanoparticles (IONPs) have been used for preparation of multifunctional thin films and membranes with numerous applications. Thus, maghemite (γ-Fe_2_O_3_) and magnetite Fe_3_O_4_ nanoparticles (NPs) are two of the most important iron oxides for food safety (packing [5], insecticide extraction [6], sensing for contaminants [7]) in adsorption and photocatalytic processes (heavy metal detection and removal [8] and degradation of organic pollutants [9,10,11,12,13,14]), electronic field (batteries and magnetic storage media [1,15]) and theranostic applications [16,17,18]. When the particle size drops below 50 nm, and especially below 20 nm, their magnetic properties change considerably. New emerging properties have been obtained as a result of the synergic action of the size and shape quantic effects on the nanostructured oxide-based materials [11,19]. Since biocompatible IONPs as quantum dots, bares or 3D nanostructures/nano-objects, are easily manipulated with precision in different environments, they represent candidates for the latest applications in medicine (targeted drug delivery, magnetic imaging, magnetic hyperthermia and thermo-ablation, bioseparation and biosensing [16]).

The porous and superporous semiconductive superparamagnetic IONPs are nowadays intensively studied for obtaining new functional composite nanostructured materials, such as multifunctional nano-adsorbents, nano-photocatalysts [11], and biomimetic and nanostructured catalytic membranes [20,21]. The maghemite (γ-Fe_2_O_3_), considered as fully oxidized magnetite, is an n-type semiconductor with a band gap energy of 2.0 eV and is a visible (Vis) domain active catalyst. Magnetite (Fe_3_O_4_) containing divalent and trivalent iron can be both an n- and p-type semiconductor and has the smallest bandgap energy (0.1 eV) and lowest resistivity among iron oxides [16].

Magnetic properties of IONPs can be adjusted by selection of the synthesis route. Despite many preparation methods (physical and chemical methods) with their advantages and limitations, obtaining IONPs with predetermined parameters (crystallinity, size, and morphology) and properties is still a challenge. For instance, the co-precipitation method, a rapid and widely used method to obtain IONPs with controlled composition and particle sizes, can trigger irregular crystal shape and agglomeration of the products [22,23,24]. Type of precursors, Fe^2+^/Fe^3+^ ratio, pH, temperature, and atmosphere are the most important parameters of the co-precipitation synthesis of IONPs. Ultrasonic assisted co-precipitation method allows better control of particle shape [24,25], while oxygen-free atmosphere is often preferred to hinder magnetite oxidation [24]. In order to control nanoparticle size, chemical stability, carrier mobility in semiconductor structures and to prevent agglomeration, coating of the IONPs has been carried out using organic or inorganic polymers, multifunctional organic and organic molecules, and carbon nanostructures [1,26,27,28,29,30,31,32]. Surface functionalization with SiO_2_ (shell) of the superparamagnetic iron oxide (core) is one of the most used and is easy to achieve and does not introduce toxic compounds [27]. Presence of Si-OH silanol groups on the surface of the magnetite nanoparticles coated with silica allows various subsequent surface functionalization, which can result in a large class of complex hybrid nanostructured compounds and applications [27,28,29]. Fe_3_O_4_/SiO_2_ core–shell nano-cubes have confirmed the ability to bind to biomolecules and to be suitable for biosensing applications [30]. The surface of the γ-Fe_2_O_3_ nanoparticles was hydrothermally modified with SiO_2_/Ag in order to absorb and subsequently remove heavy metal ions [31]. Magnetic core–shell dendritic silica Fe_3_O_4_@SiO_2_@Dendritic-SiO_2_ composite with excellent catalytic activity and convenient recovery was prepared by an oil–water biphasic stratification coating [33]. Double cover of SiO_2_/TiO_2_ was also applied to the IONPs and enhanced photocatalytic MB degradation activity was reported for Fe_3_O_4_/SiO_2_/TiO_2_ core-shell nanoparticles under UV light irradiation [9]. Curcumin-Fe_3_O_4_/SiO_2_/ZnO nanocomposites were prepared as an anticancer drug delivery system [34].

In recent years, due to the need to expand photocatalytic processes for advanced wastewater treatment, new structured and heterostructured thin film semiconductor photocatalytic systems have been proposed for direct use of visible (Vis)- or solar-active catalysts [35,36,37,38,39]. Thin-film (photo)catalysts are an emerging field with considerable potential to be used in the industries because their synthesis is suitable and economic for handling and adapting the catalytic materials’ form for the reactors, in their recycling and recovery [35]. Beside TiO_2_ and ZnO-based multilayered thin film [36,37], α-Fe_2_O_3_ thin films on Si(100) and SrTiO_3_ substrates thin films were investigated for photocatalytic dye degradation [38,39]. The sol-gel obtain SiO_2_ cover of the Fe_3_O_4_@SiO_2_@ZnO photocatalysts, used for the degradation of Acid Blue 161 dye, played a double role, e.g., preventing magnetite agglomeration and lowering of the ZnO bandgap (3.37 eV) [40]. The efficiency of the low-cost processing sol-gel method for obtaining and/or surface modification of IONPs might be still limited [28]. Therefore, improving the existing methods as well as discovering of new ones is required.

This work aims to use co-precipitation and the ultrasonic-assisted sol-gel method to obtain iron oxide nanoparticles functionalized with SiO_2_ and SiO_2_/ZnO. The novelty of this study consists of the investigation of the combined effect of the magnetic core co-precipitation temperature (80 and 95 °C) in air and ZnO-doping (commercial ZnO nanoparticles) of the silica shell on the photocatalytic and nano-sorption properties of the resulting composite nanoparticles, for compatibility with integration in thin films to expand photocatalytic processes for advanced wastewater treatment at the industrial level.

## 2. Materials and Methods

### 2.1. Reagents

The reagents used in the synthesis of nanoparticles were ferric chloride hexahydrate (FeCl_3_·6H_2_O), ferrous sulfate heptahydrate (FeSO_4_∙7H_2_O), tetraethyl orthosilicate (TEOS), and sodium hydroxide (NaOH) with analytical quality, without further purification, and they were purchased from Sigma-Aldrich. The 50 nm diameter ZnO nanoparticles were purchased from Merck Romania SRL (Bucharest).

### 2.2. Methods

The investigated MNPs were obtained in a two-step approach from solution. In the first step, iron oxide nanoparticles were synthetized by a simple co-precipitation and subsequently used in a hybrid sol-gel sonochemical method to obtain surface-functionalized core-shell composite IONPs. Aqueous solution of Fe(III):Fe(II), (2:1) atomic ratio was hydrolyzed in a sodium hydroxide solution (0.25M) at 95 (M1 samples) and 80 °C (M2 samples). Unlike Jing-Fu Liu’s [8] or other literature protocols, no additives were added during the synthesis in order to control the size of the nanoparticles. The as-obtained magnetic coprecipitate was isothermally aged in the mother solution (pH 11.5–12) under stirring for 60 min. In the second step, two series of derivate core-shell samples were prepared by a hybrid sol-gel sonochemical method using, separately, the M1 and M2 nanoparticles as magnetic core and TEOS or TEOS with ZnO nanoparticles (NPs) as a source for silica, SiO_2_, (S1) and ZnO-doped SiO_2_ shells (S2), following a protocol adapted from Zhao et al. [29]. Thus, the previously obtained iron oxide nanoparticles (M1 or M2) were dispersed in ethanol (ultrasonic bath), followed by water and ammonia solution added to reach a pH of ~9, under continuous sonication for 30 min. Subsequently, an appropriate volume of TEOS was added to ensure the 1:1 [Fe_3_O_4_]: [SiO_2_] molar ratio. The resulting samples were aged under five-hour stirring and then were separated by centrifugation, washed several times with ethanol, and dried at 65 °C in air for 2 h, i.e., M(1/2)-S1. For the preparation of M(1/2)-S2 core-shell MNPs, commercial ZnO NPs (3 wt.% with respect to the weight of used M1 and M2 powders) previously dispersed into ethanol were added, just after addition of TEOS reagent.

The obtained iron oxide and iron oxide-silica MNPs isopropanol dispersions (30 mg/2 mL) were used for thin film deposition (three layers) on glass substrate (2.5 × 2.5 cm) by spin-coating (500 rpm) [41]. The as-deposed films were heat plate dried at 90 °C for 10 min. The obtained films were denominated FM(1/2)-S(1/2).

### 2.3. Equipments

The surface morphology of the MNPs were investigated by scanning electron microscopy (SEM) using a JEOL JSM-7500F/FA microscope from Peabody, Massachusetts, JOEL Ltd. USA. Preparation of MNPs samples for SEM examination consisted of air drying a drop of alcoholic dispersion of the nanoparticles (into an ultrasonic bath) onto a glass substrate and coated with a 5 nm-thick Au layer by sputtering.

X-ray diffraction patterns were recorded using Rigaku’s Ultima IV diffractometer in parallel beam geometry, using Cu Kα radiation (λ = 1.5406 Å), and graphite monochromator operating at 40 kV and 30 mA. The signals were collected from 10° to 80° with a step size of 0.02° and a scan speed of 2° min^−1^. Phase identification was performed using Rigaku’s PDXL software, connected to ICDD PDF-2 database. The lattice constants were refined using Whole Powder Pattern Fitting (WPPF). The average crystallite size was calculated from the (311) diffraction line using Scherrer’s equation:

D = kλ/(β·cosθ)(1)

where k = 0.90, λ is the wavelength of X-ray, β is full width at the half maximum (FWHM) of the peak is the diffraction angle [42,43,44].

UV resonance Raman spectroscopy has been shown to be a powerful technique for the investigation of iron-based materials usually supported on silica materials [45]. UV-Raman spectra were collected on the Fe-containing powders by means of a LABRam HR800 spectrometer (Horiba France SAS, Palaiseau, France). The exciting He-Cd laser of 325 nm (Kimmon Koha Co LTD, Tokyo, Japan) was focused on samples through an Olympus microscope objective of 40× NUV/0.47. The laser power was kept as low as possible to prevent sample heating. At least three spectra were collected for each sample.

Nitrogen adsorption-desorption isotherms at 77 K were recorded on a Micromeritics ASAP 2020 analyzer (Norcross, GA, USA). The samples were degassed at 90 °C for 5 h under vacuum before analysis. Specific surface areas (SBET) were calculated according to the Brunauer–Emmett–Teller (BET) equation, using adsorption data in the relative pressure range between 0.05 and 0.30. The total pore volume (Vtotal) was estimated from the amount adsorbed at the relative pressure of 0.99. The pore size distribution curves were obtained from the desorption data using the BJH (Barrett-Joyner-Halenda) model [46].

Magnetic properties were assessed at room temperature on Lake Shore’s fully integrated Vibrating Sample Magnetometer system 7404 (VSM) (Westerville, OH, USA). The corresponding thin films deposited on the glass substrates were characterized for magneto-optical measurements by magnetic circular dichroism (MCD) using a JASCO 815 spectrometer equipped with a static magnet of 1.5 T and circular polarized light at a rate of 50 kHz. The MCD value was measured in the spectral range 700–300 nm at the temperature 300 K [47].

The photocatalytic properties of the synthesized samples (40 mg) were conducted by UV-Vis spectroscopic monitoring (SPECORD 210 PLUS Double-beam Spectrophotometer from Analytik Jena, Jena, Germany, equipped with a WinASPECT PLUS software Version: 4.3.0.0) of the degradation of methylene blue (MB) dye solution (25 mL, 5 mg/L) under UV irradiation (254 nm), using a mercury UV lamp at 100 KW. The desorption test carried out in deionized water with powders separated from MB solution after 2 h exposure at UV light and 2 days rest in demi-darkness. The resulting solutions were measured for recovered MB by optical absorption in the range of 500–750 nm.

## 3. Results and Discussion

### 3.1. Morphology and Structure

**Figure 1:**
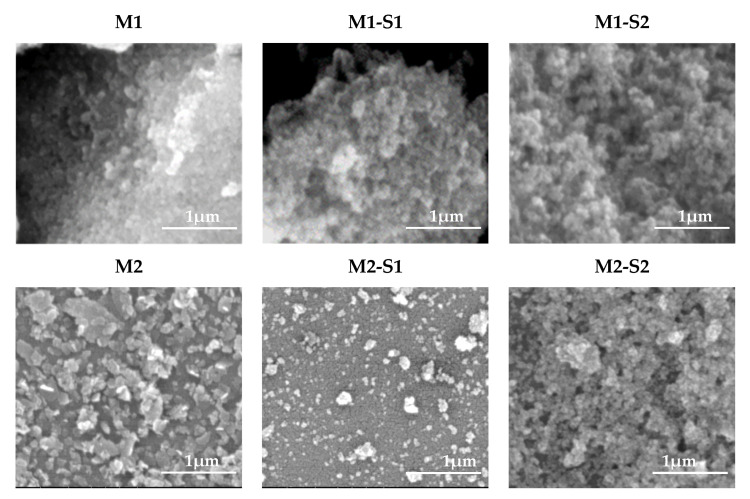
Surface SEM images of iron oxide and the corresponding core-shell composites nanoparticles.

The top-view SEM morphology of the investigated samples are shown in Figure 1. Nanoparticles size for samples M1 and M1-S1/2 ranges from 26–33, 22–29 and 16–26 nm, respectively, while for M2 and M2-S1/2 it varies from 16–24 to 12–20, and 16–32 nm, respectively. As expected [27,28,29], smaller particles depicted in the M1-S(1/2) samples point out that SiO_2_ and Zn-doped SiO_2_ covers prevented agglomeration of the M1 cores. The same reasoning is valid for M2-S1 sample. However, the M2-S2 nanoparticles seem to be alike M2 ones. Self-assembly of MNPs in short strings but also in nanoplatelets or 3D nanoaggregates can be observed.

**Figure 2:**
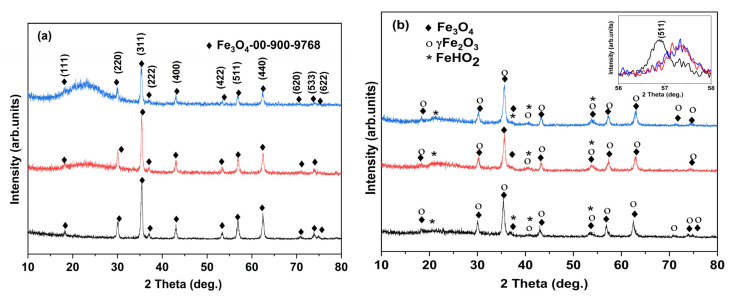
XRD patterns of powders in the (**a**) M1 series and (**b**) M2 series: M1 and M2 (black diffractograms) and corresponding core-shell composite MNPs, M(1/2)-S1 (red), and M(1/2)-S2 (blue).

Figure 2 illustrates the XRD patterns of the investigated samples. For the first series (Figure 2a), the patterns show diffraction lines corresponding to magnetite, Fe_3_O_4_, as the major phase and maghemite (about 20%) (JCPDS no. 01-076-1849) [42,43,44]. The average crystallite size calculated using the (311) plane was 22 nm (M1) 29 m, (M1-S1) and 23 nm (M1-S2). The lattice parameters calculated from the XRD data (Table 1) are in good agreement with the values reported in the standard JCPDS no. 01-076-1849, showing a slight increase for SiO_2_ shell functionalized samples.

In the case of the second series (M2, M2-S(1/2)) samples, a significant variation of the lattice parameters values can be noticed (Table 1). The advanced processing of the XRD (Figure 2b)-highlighted phase composition changes, starting from the M2 sample, i.e., the presence of a mixture of magnetite and maghemite (γ-Fe_2_O_3_) phases, the latter consistently representing the majority phase. In the synthesis of core-shell NPs (M2-S1 and M2-S2), the latter (maghemite) increases to ~72 and ~76%, respectively, Fe_3_O_4_ representing only 15 and 11%, respectively (Table 1). The average crystallite size calculated using the (311) plane was 17 nm (M2) and 18 nm (M2-S1, M2-S2). The lattice parameters calculated from the XRD data are presented in Table 1 (JCPDS no. 01-076-1849) [42].

A broad diffraction line can be observed at 23.15°, especially for the core-shell composite samples, corresponding to vitreous SiO_2_ shell.

**Figure 3:**
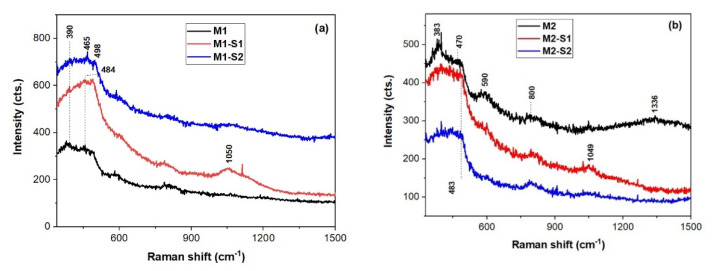
UV-Raman spectra of powders in the (**a**) M1 series and (**b**) M2 series: M1 and M2 (black spectra) and corresponding core-shell composite MNPs, M(1/2)-S1 (red), and M(1/2)-S2 (blue).

Raman spectra of the investigated samples are shown in Figure 3. The band located at about 380 cm^−1^ for M1, M1-S1 and M2, powders might belong to goethite (α-FeO(OH)), lepidocrocite (γ-FeO(OH)) [48]. Heating goethite at about 700 °C converts it into hematite (α-Fe_2_O_3_), which is a strong Raman scatterer. However, local heating induced by laser cannot reach this temperature. On the other hand, maghemite (γ-Fe_2_O_3_), a weak Raman scatterer, can be obtained by heating lepidocrocite at about 400 °C. The wide band peaking up within 1300–1336 cm^−1^ belongs to hematite as well as maghemite. Hence, a mixture of maghemite and hematite can coexist in non-functionalized samples M1 and M2 and functionalized M1-S1 sample. Except preparation methods and laser irradiation parameters (duration and power) during recording Raman spectra, particle size is another factor influencing the presence of both hematite and maghemite [49]. It is known that maghemite transforms irreversibly in hematite at elevated temperatures [50]. Moreover, another weak Raman scatterer, magnetite (Fe_3_O_4_), with a characteristic band at about 670 cm^−1^, can oxidize to maghemite and further to hematite under laser irradiation [51]. Since it is difficult to ascribe the few relatively weak and wide bands at ~380, 470, ~640, and ~1330 cm^−1^ to a certain iron oxide, they should be correlated with XRD information.

Moreover, functionalized samples M(1/2)-S(1/2), show spectral features of the SiO_2_ at 480, ~600 (shoulder), ~800, 970, and 1049 cm^−1^ due to defect bands O_3_SiOH (D_4_) [52] and three-membered SiO_4_ rings (D_3_), stretching vibrations of Si-OH, and Si-O-Si bonds. The main 1LO band of ZnO nanoparticles under UV excitation at about 570 cm^−1^ [53] might be obscured by the D_3_ band of the SiO_2_ in the Raman spectra of the SiO_2_-ZnO functionalized iron oxide nanoparticle in Figure 3. This proves successful covering of iron oxide particle by SiO_2_ in so called core-shell nanoparticles [54].

### 3.2. Textural Analysis

**Figure 4:**
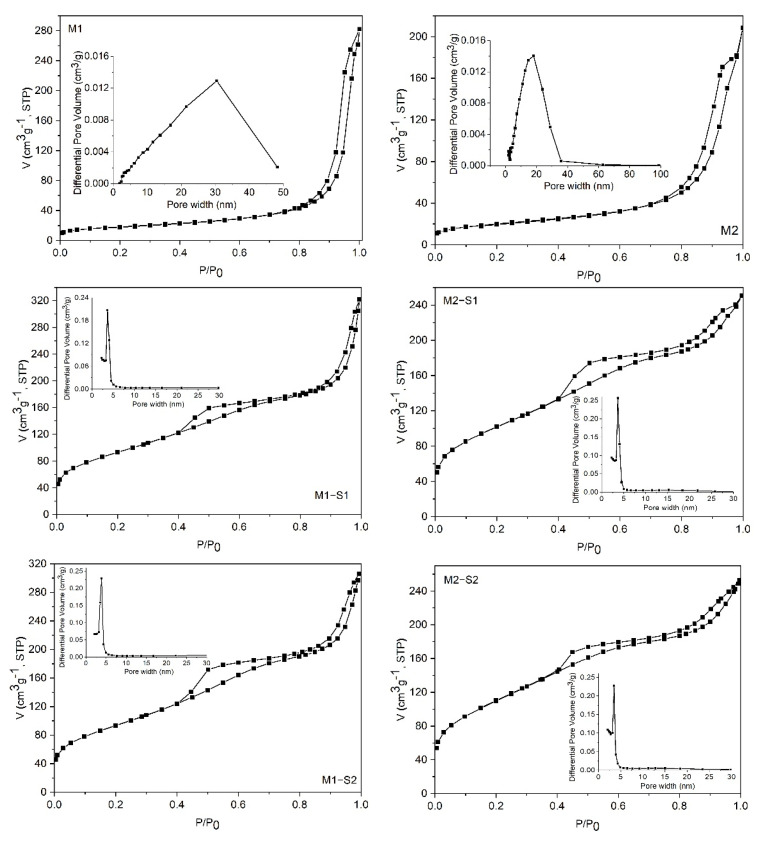
N_2_ adsorption–desorption isotherms and pore size distributions (inset of the figures) of the investigated samples.

The textural features of the samples were investigated by N_2_ physisorption measurements. All isotherms (Figure 4) are of type IV [46], typical for mesoporous materials, but the shape of the isotherms and the hysteresis loops are different for simple iron oxides (M1 and M2) compared to the corresponding silica-containing composites. In the case of M1 and M2 samples, the adsorption isotherms exhibit capillary condensation in their high relative pressures regions (p/p0 > 0.8), which indicates the existence of large mesopores constituted mainly by the interstices between the nanoparticles. The pore size distributions (inset of the figures) confirm this observation. For all core-shell composites, the isotherms retain the footprint of the incorporated iron oxide in the high-pressure region but exhibit capillary condensation starting from lower relative pressures of about 0.4. This behavior is attributed to the silica layer on the surface of the iron oxide nanoparticles. The hysteresis loops are a combination of H2 and H3 types, which indicate the existence of two types of mesopores. Accordingly, the pore size distribution graphs for all composites display a predominant type of mesopores ranging from 2 to 6 nm and a second one comprising the interparticle voids. The textural parameters (BET surface area and total pore volume) are listed in Table 1. One can note a significant increase of total pore volumes and BET surface areas of the composites compared to those of the corresponding iron oxides (M1/M2).

### 3.3. Magnetic Properties

**Figure 5:**
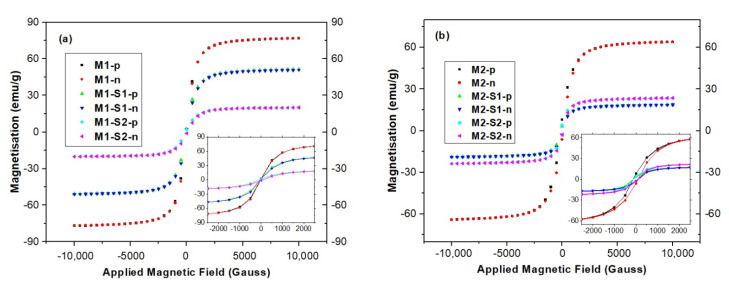
Magnetic field dependence of magnetization of the (**a**) M1 and M1-S(1/2); and (**b**) M2 and M2-S(1/2) powders. Insets represent detailed representations within ±2500 Gauss range.

Magnetic properties of the samples were investigated by vibrating sample magnetometry (VSM). The field-dependent magnetization curves at room temperature display a superparamagnetic behavior for all the synthesized samples, without hysteresis loop (Hc about 0.5–1.5 Oe) and almost zero remanent magnetization (Figure 5).

The zero values of remanence magnetization and coercivity observed on the hysteresis curves indicate that all samples are superparamagnetic. The calculated saturation magnetization (Ms) and µ/k resulted from nonlinear regression of measured data with Langevin function. According to data in Table 1, saturation magnetization (M_S_) values are highly dependent on the type of iron oxide present in the samples, processing temperature, textural parameters, and the type of the core-shell system formed. High saturation values around 70 emu/g show strong induced magnetization behavior of iron oxide samples (M1 and M2) [29]. These values slightly diminish as synthesis temperature lowers. Bulk magnetite (Fe_3_O_4_) and maghemite (γ-Fe_2_O_3_) have M_S_ values of 90 and 76 emu/g, respectively [55,56], while the corresponding SiO_2_-functionalized nanoparticles encountered smaller values [56,57,58]. The Ms value for the M2 containing 61.16% γ-Fe_2_O_3_ and 37.08% Fe_3_O_4_ is very close to the one reported in literature for commercial maghemite with 13 nm mean-sized particles [57]. After coating with SiO_2_ layer, the saturation magnetization gradually decreased to 32–35 and 20–25 emu/g for M1-S1/2 and M2-S1/2 samples, respectively (Table 1), as a result of decreasing subsequences in magnetism and quenching of surface magnetic moments [16]. These values fully met the requirements as superparamagnetically active material [25]. Slight increases of magnetization were recorded when ZnO was used for preparation of the SiO_2_-shell e.g., M(1/2)-S2, in comparison with the silica-iron oxide samples, M(1/2)-S1.

**Figure 6:**
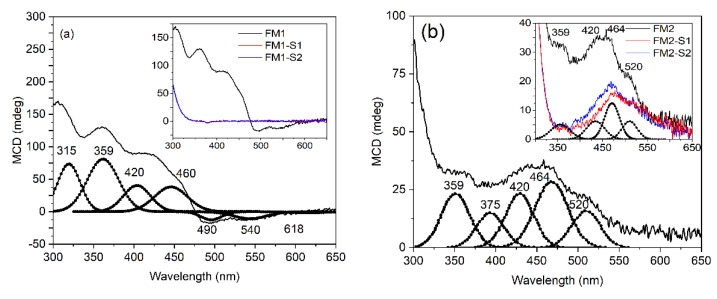
Fitted MCD spectra of: (**a**) FM1, (FM1-S1 and FM1-S2 in the inlet) and (**b**) FM2 sample, (FM2-S1 and FM2-S2 in the inlet) magnetic thin films deposed on glass substrate.

The magneto-optical properties of the core magnetic nanoparticles (M1 and M2) and those obtained after SiO_2_-shell deposition through the sol-gel processing method (M1-S1, M1-S2, M2-S1 and M2-S2), were investigated using the circular magnetic dichroism (MCD) technique. This technique allows for the identification of paramagnetic and diamagnetic properties of Fe_3_O_4_ and Fe_2_O_3_ thin films under different environments through their specific characteristics resulting from positive and negative absorptions under magnetic field [59]. The magneto-optical properties of the FM1 core and their associated FM1-S1 and FM1-S2 core-shell thin films are revealed from the Figure 6a. The M1 core sample presents a series of negative and positive peaks starting with 618 nm, suggesting a paramagnetic behavior assigned to Fe_3_O_4_. As can be seen, after core-shell formation, the paramagnetic behaviors of the spinel Fe_3_O_4_ are hindered in both FM1-S1, FM1-S2 (inlet).

The magneto-optical measurements on FM2 core samples and their associated core-shell structures FM2-S1 and FM2-S2 (detailed in Figure 6b) suggest the presence of Fe_2_O_3_ as the majority phase. The core samples exhibit three main peaks centered at 520 nm, 464 nm, and 375 nm associated with γ-Fe_2_O_3_ while some positive peaks centered at 420 and 359 nm may be associated with Fe_3_O_4_.

Magnetite (Fe_3_O_4_) crystallizes in the inverse spinel structure having one-third of Fe^3+^ (t2g^3^eg^2^, S = 5/2) ions surrounded by the four oxygen ions in the tetrahedral symmetry (A-site) while the other two-thirds of the Fe is a combination of Fe^2+^ (t2g^4^eg^2^, S = 2) and Fe^3+^ (t2g^3^eg^2^, S = 5/2) ions surrounded by six oxygen atoms in the octahedral symmetry (B-site). In this context, the Fe^3+^ ions give paramagnetic behaviors due to the unpaired electrons in orbitals, while the Fe^2+^ in the low spin configuration possesses diamagnetic behaviors strongly dependent on the surrounding crystal field. The main absorption bands are connected with the d–d transitions influenced by the lattice distortions, but some of them are mediated by the surrounded oxygen atoms as intersite but also as intrasite through the overlapping p−d orbital states. The first observed negative band at 618 nm (2 eV) is assigned to ^6^A_1g_(^6^S)−^4^T_1g_(^4^G) of Fe_3_O_4_ in the octahedral symmetry due to an intervalence charge transfer IVCT, while the 540 nm (2.3 eV) is assigned to optical transition across the valence gap of the spin-majority (Fe^3+^) between the B-site (e_g_)↑ and A-site (e_g_, t_2g_)↑ [59]. The third band centered at 460 nm (2.69 eV) overlaps two transitions due to the intersystem charge transfer as a mixture of two transitions [Fe^3+^]_eg_ → [Fe^2+^]_e_; [Fe^3+^]_t2_ → [Fe^2+^]_t2g_ as intersublattice charge transfer [60]. The band from 420 nm (2.95 eV) is assigned to Fe^2+^ because of intervalence charge transfer. The next two bands from 359 nm (3.46 eV) and 315 nm (3.93 eV) are given by intersublattice charge-transfers between the octahedral and tetrahedral configurations. The maghemite core nanoparticles (γ-Fe_2_O_3_) give different diamagnetic transitions centered at 520 nm (2.38 eV) and 464 nm (2.67 eV), with the Fe^3+^ in the tetrahedral respectively octahedral positions, while the 375 nm is a ligand-to-metal charge transfer from oxygen ions to Fe^3+^ in the octahedral position [61,62,63]. The other two bands from 420 nm and 359 nm are assigned to Fe_3_O_4_ as already described.

The FM1 film samples exhibit mainly Fe_3_O_4_ structures and their core-shell FM1-S1 and FM1-S2 films obtained by SiO_2_ and ZnO shells hindered the magneto-optical properties of the Fe ions in the A or B sites of the spine structures. The main phase is the magnetite structure in good agreement with the XRD measurements.

The FM2 films suggest a majority phase of γ-Fe_2_O_3_ given by the d–d charge transfer of Fe^3+^ in the tetrahedral respectively octahedral positions and the 375 nm ligand-to-metal charge transfer from oxygen ions to Fe^3+^ in the octahedral position. Furthermore, besides the γ-Fe_2_O_3_ majority phase, some intersublattice charge-transfers between the octahedral and tetrahedral configurations in the ultraviolet range are assigned to the Fe_3_O_4_ spinel structures as minority phase in good accordance with the XRD results.

### 3.4. Nanosorption and Photocatalytic Activities

#### 3.4.1. Sorption Kinetics

**Figure 7:**
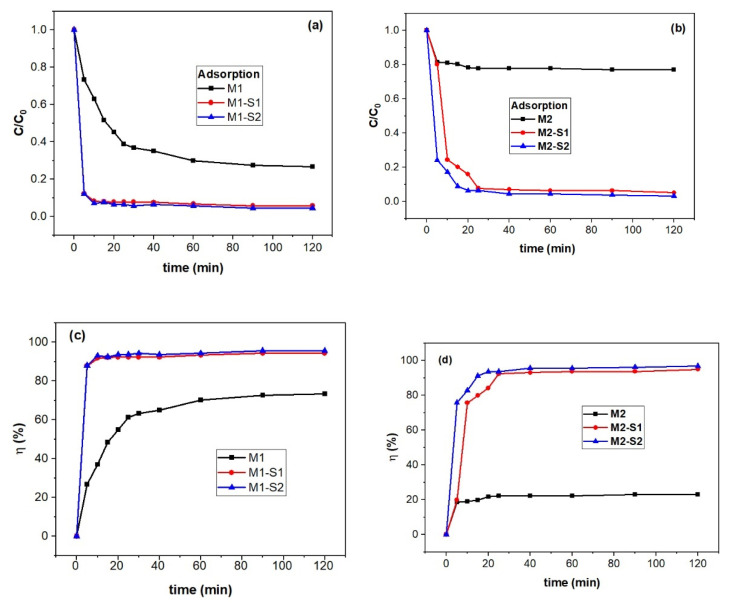
Dark adsorption versus time (**a**,**b**) and adsorption efficiency (**c**,**d**) of methylene blue (MB) on M(1/2)-S(1/2) samples (c(MB) = 5 mg/L, V = 25 mL of MB solution and w(M(1/2)-S(1/2) = 0.040 g at room temperature).

The photocatalysis in heterogeneous systems are complex processes [64]. One of the important stages of the photocatalytic processes is reactant adsorption on the catalyst surface. Hence adsorption tests in darkness of the MB on the M(1/2)-S(1/2) samples were carried out while adsorption efficiency was calculated [9,65] and illustrated along with residual MB concentration in Figure 7. The latter data are spectroscopically derived from the absorbance band at 665 nm of the MB solution in the presence of M1, M2 powders and the corresponding core-shell nanoparticles after 120 min in darkness (Figure S1).

The adsorption efficiency, η, was calculated with the following equation:

(2) $$\eta =\frac{C_{0}-C_{t}}{C_{o}}\times 100\%$$

where C_0_ and C_t_ are the initial concentration of MB (mg/L) before adsorption and at time t (min) of sorption process. According to data in Figure 7c,d, an adsorption efficiency ~70% MB was observed for the higher processed IONPs M1 sample at 95 °C (0.393 cm^3^∙g^−1^ in Table 1) and only ~21% for the 80 °C counterpart (M2, 0.322 cm^3^∙g^−1^).

A kinetic study was carried out to obtain information on the type of interaction between the active centers of adsorbent and methylene blue. Thus, physical interaction of active centers of an adsorbent and adsorbate is well described by a pseudo-first-order (PFO) model of kinetics while the pseudo-second-order (PSO) kinetics refers to the chemisorbed adsorbate, e.g., surface-interaction kinetics models. A third model developed for aqueous solutions–porous solid systems [65] assumes instantaneous dye-adsorbent interaction relative to intraparticle diffusion (ID) and hence, kinetics is ruled by diffusion. A Weber–Morris equation is used to obtain the duration of diffusion steps and if the absorption process is controlled by diffusion [65].

In the case of the M(1/2)-S(1/2) samples, pseudo-second-order (PSO) and intraparticle diffusion (ID) were applied as adsorption kinetics of MB (see Table 2). The PSO equation is the following:

(3) $$\frac{dq}{dt}=k_{2}{\left(q_{e}-q_{t}\right)}^{2}$$

where k_2,_ q_e_ and q_t_ are the rate constant for the PSO adsorption process, amounts of MB (mg/g) adsorbed onto the catalysts at equilibrium and time t, respectively.

**Figure 8:**
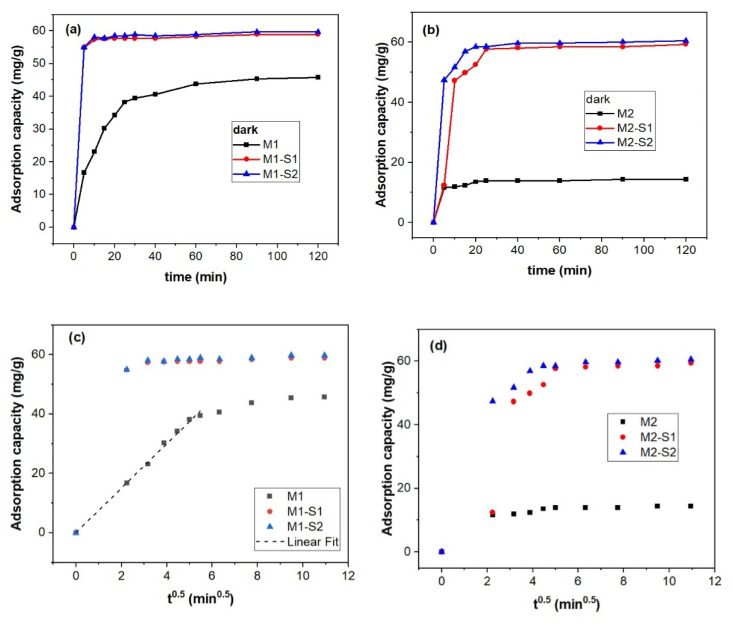
Adsorption capacity (q_t_) against contact time (**a**,**b**), ID model (**c**,**d**) of the M(1/2)-S(1/1) for MB (c(MB) = 5 mg/L, V = 25 mL of MB solution, and w(M(1/2)-S(1/2) = 0.040 g at room temperature).

Figure 8 illustrates adsorption capacity (q_t_) against contact time (t) and t^0.5^ for deriving the kinetic information. Bigger pore volume of the M1 sample obtained at 95 °C (0.393 cm^3^∙g^−1^ in Table 1) enables three times higher adsorption capacity of MB (~45 mg/g in Figure 8a) than the M2 sample (0.322 cm^3^∙g^−1^). This discrepancy almost vanishes for the core-shell samples M1-S1/2 si M2-S1/2 (q_t_ of 59–61 mg/g in Figure 8a,b).

According to the data in Table 2 and Figure 8b,c, the adsorption of MB molecules on the investigated MNPs is controlled by intraparticle diffusion within the 0–33 min range. Faster adsorption was recorded for the M1 series in contrast with the M2 series as noticeable from the k_id_ values.

Core-shell functionalization triggers the enhancement of the specific surface and pore volumes by 6 and 20–25% times, respectively. These modifications are slightly smaller for the MNPs specific surface of the MNPs obtained at 95 °C, since its bigger crystallites were self-assembling in aggregates with meso- and micropores. Instead, the MNPs synthesized at 80 °C having smaller pore volumes are self-assembled in more compact aggregates as confirmed by SEM and BET information.

#### 3.4.2. Photocatalytic Activity and Stability

The easy and friendly photodegradation of organic compound dyes soluble in water, like MB, in the presence of transition-metal oxide (TMO) single- or multi-component photocatalyst is an oxidative process. The break-down of the complex molecule into less- or nontoxic simple molecules and ions (CO_2_, H_2_O, SO_4_^2−^, NH_4_^+^ etc.) does not result from a direct simple redox reaction between the semiconductive oxide nanoparticles and the MB molecules, the electron transfer it is done through a series of intermediaries, within a complex multistep process mechanism [66].

Under light exposure, the TMO photocatalyst is activated by absorbing photons with higher energy than its bandgap, generating an electron-hole (é-h^+^) pair. These charge-carrier particles, in suitable conditions to avoid their recombination, interact with the molecules or ions in the reaction medium, generating a series of highly reactive oxidative radicals and/or molecules (ROS species) [67]:

(4) $$O_{2}+é\;\to \;\cdot O_{2}^{-}$$

O_2_ + 2H^+^ + 2é → ∙H_2_O_2_ (5)

h^+^ + H_2_O → ∙∙HO^0^ + H^+^ (6)

The key reactive species are holes (h^+^), hydroxyl (HO^0^) and superoxide (^•^O^−^_2_**)** radicals. Thus, the essential condition for the efficient operation of the semiconductor photocatalyst is to prevent the recombination of the two types of charge carriers that generate these radicals in the reaction medium. Modification and functionalization of the photocatalyst surface, including core-shell nanostructuring, is an important approach to tuning photocatalytic properties. Thus, the high enhancement in photocatalytic activity of SiO_2_ functionalized IONPs is explained by the effect of the silica shell on reducing the bandgap energy and the electron-hole recombination [67].

**Figure 9:**
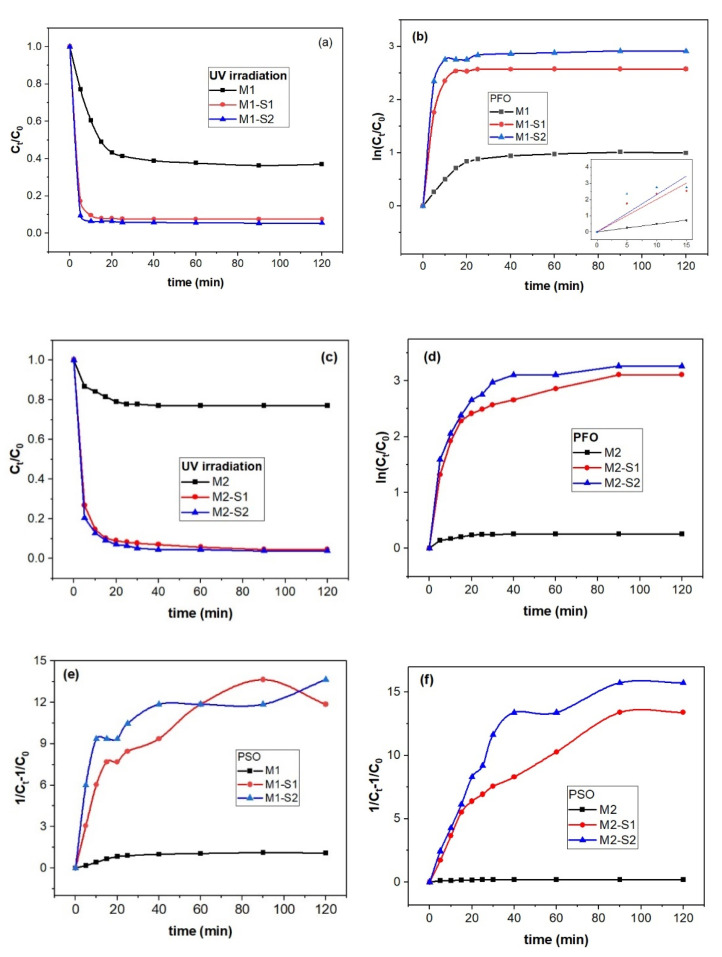
Photocatalytic degradation of MB on M1, M2 and M(1/2)-S(1/2) nanocatalysts via UV-Vis spectrophotometry (**a**): Plots of PFO (**b**,**d**) and PSO model (**e**,**f**) for MB degradation (**c**)(MB) = 5 mg/L, V = 25 mL of MB solution, and w(M(1/2)−S(1/2) = 0.040 mg at room temperature).

The light radiation wavelength, time irradiation, initial dye concentration, photocatalyst composition, electronic structure and morphology are the main parameters to control rate and mechanism of photodegradation of dyes and organic compounds [68,69]. Figure 9 shows experimental data of the photocatalytic degradation of MB under UV irradiation. To identify the mechanism and stage determined by the rate of MB degradation in the presence of synthesized photocatalytic nanoparticles, these data were fitted with pseudo-first-order (PFO) and pseudo-second-order (PSO) kinetic models [68].

As depicted in Figure 9, degradation of MB on M(1/2)-S(1/2) takes place in two steps. A first stage up to 15–30 min. with a steeper slope is followed by a slower process. Maximum removal of MB increases in succession M1 (~62.4%) < M1-S1 (~92.4%) < M1-S2 (94.4%) after 60 min (Figure 9a). The k_1_ value for the M1 sample (0.048 min.^−1^) is similar to the one reported in literature (0.046 min.^−1^) for Fe_3_O_4_ at 303 K (MB 40 mg/L) [69]. Ten times faster degradation first-order rate values, k1, than the one reported by Dangher [32] for Fe_3_O_4_-SiO_2_ photocatalyst, were obtained for M1-S1 (0.20105 min.^−1^) and M1-S2 (0.23024 min.^−1^ in Table 3). A second order process seems more adequate for the degradation of samples from M2 series. M1 shows faster degradation of MB than the M2 sample while the corresponding core-shell samples (M1-S1/2 and M2-S1/2) caused three–four times faster degradation of MB. In conclusion, while first order kinetics are suitable to describe the degradation process of M1 series, a second-order process seems more adequate for M2 series.

**Figure 10:**
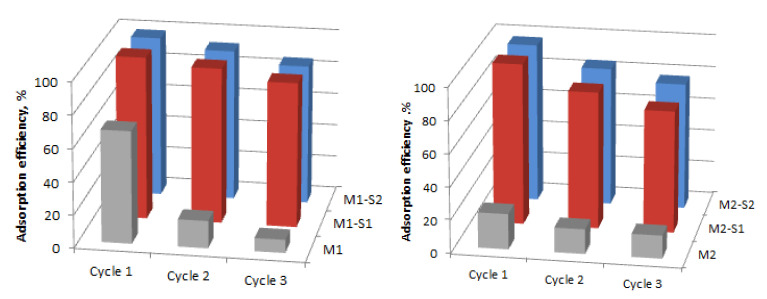
Adsorption efficiencies of the M1, M2 and M(1/2)-S(1/2) catalysts for MB adsorption under darkness (120 min).

**Figure 11:**
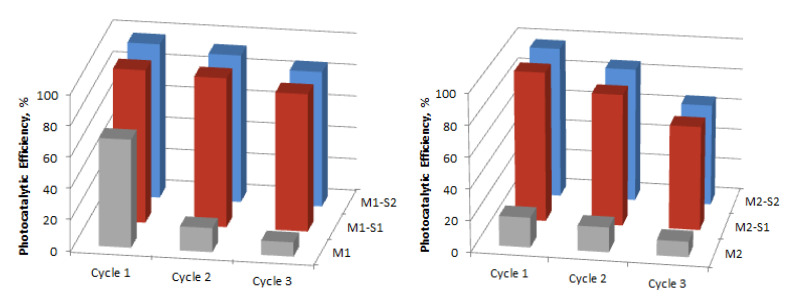
Reusability efficiencies of the M1, M2 and M(1/2)-S(1/2) catalysts for MB degradation under UV irradiation (120 min).

The stability of the nanocatalysts is very important for further use of these materials. The sorption and photocatalytic performances of the recycled nanoparticles were evaluated during three successive cycles, under darkness and UV irradiation, respectively, for 120 min. The variation of the adsorption efficiency and photocatalytic degradation efficiency are presented in Figure 10 and Figure 11. Regarding the behavior of the core-shell particles from the M1-S1/2 series, the adsorption efficiency of the recycled IONPs reduced from about 99–98% to 95–93% (Cycle 2) and 88–85% (Cycle 3). The decreases are slightly more important in the M2-S1/2 series, namely, from 98–96 to 85–84% (cycle 2) and 77–75% (Cycle 3). Significant decreases in the efficiency of sorption and photocatalysis in the case of M1 and M2 samples, but also after the three cycles, can be attributed to the increase of the positive surface charging of IONPs leading to agglomeration into clusters [1].

Slightly 1–2% lower values of photocatalytic efficiency (Figure 11) compared to those of equilibrium adsorbency (Figure 10) may indicate, in addition to some morphological changes, possible changes in semiconductor structure. As depicted in Figure 10 and Figure 11, after three recycles the best stability was recorded for the M1-S1 sample and the worse one belongs to the M1-S2 sample. The effect of the disposal procedure for the remanent adsorbed MB molecules and/or the degradation products from the reused catalyst surface also has to be investigated further. Among the core-shell nanocatalysts, ZnO-doped samples show weaker stability in contrast with the ones with single SiO_2_ covers. This behavior points out slightly lower stability of the ZnO-doped SiO_2_ covers among the functionalized samples.

In order to demonstrate whether the process of MB solution discoloration is truly catalytic oxidation, after reaching the adsorption–desorption equilibrium, the used MNPs were separated from the solution and tested for the leaching of dye molecules in water. The resulting solutions, except for the M1 sample, were colorless (Figure S2) and showed no absorbance peak when examined by visible (VIS) spectrophotometry (Figure S3). These results confirm that for these samples, the discoloration of MB solutions is not a reversible adsorption phenomenon, but an irreversible process of oxidative photocatalytic degradation. In the case of sample M1, with adsorption efficiency in the dark three times higher than M2 (~70%, Figure 7c), the MB leakage solution coloration (Figure S2) can be explained by that the adsorption efficiency (~70%, Figure 7c) in pores exceeds the photocatalytic efficiency (~58%, cycle 1 in Figure 11), taking place only a partial degradation of the MB molecules adsorbed by the catalyst. At the same time, M2 samples showed very closed adsorption efficiency (20%, Figure 7d) and photodegradation efficiency (~19%, Figure 11). These samples behave quite similar, and the adsorbed molecules are totally degraded. Taking into consideration that the magnetic phases of M2-based nanoparticles is maghemite (a very small bandgap energy semiconductor, Eg, of −0.1eV, with small and very good photocatalytic activity in semi-darkness and visible light, respectively [16]), one can explain the colorless rinsed water (Figure S2).

**Figure 12:**
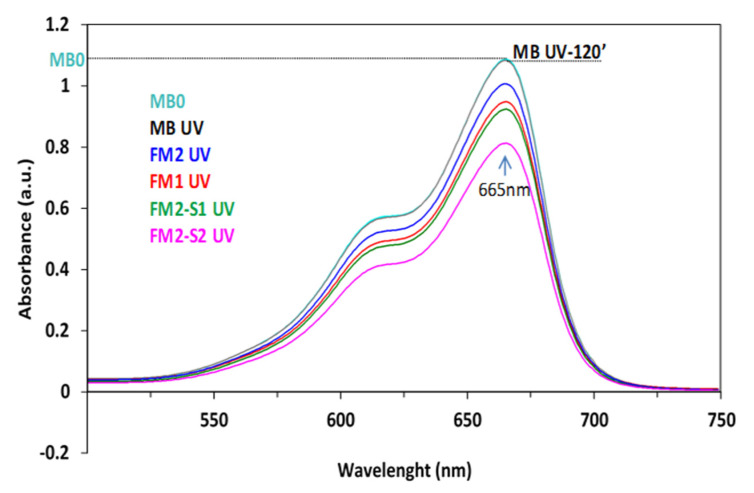
Absorbance spectra of the MB solution, before (MB0) and after 120 min. exposure to UV, without (MB UV) or in the presence of the investigated thin films (Inset-Digital photo of tested films).

Figure 12 shows the absorbance spectra of the MB solution after 120 min exposure to UV irradiation in the presence of iron oxide thin films (FM1 and FM2) and corresponding core-shell FM2-S1/2-based thin films.

The decrease in the intensity of the main absorption peak in the visible (665 nm) indicates the obvious photocatalytic activity of all the tested films. This increases for samples with core-shell samples, the increase being more consistent for that in which the shell is doped with ZnO (FM2-S2).

## 4. Conclusions

The paper presents a study of obtaining magnetic nanoparticles compatible with integration in thin films to expand photocatalytic processes for advanced wastewater treatment at the industrial level. Core-shell iron oxide nanomaterials were synthetized by co-precipitation at 80 and 95 °C in air and ultrasonic-assisted sol-gel methods. UV-Raman spectroscopy proved successful functionalization of the iron oxide nanomaterials. All the magnetic nanopowder samples presented here are superparamagnetic. Magnetite (~80%, crystallite size of 22Å, Ms > 70 emu/g) was the main phase of iron oxide depicted in the coprecipitates sample in air at higher temperature (95 °C) while the maghemite phase with modified structural parameters prevails in the 80 °C coprecipitated sample. The biggest values of magnetization were derived for the iron oxide nanoparticles. Surface functionalization with SiO_2_ of iron oxide nanoparticles triggers diminishing by 50–60% of the Ms along with partial transformation of magnetite into maghemite.

The MB adsorption capacity increases from ~15 to 45 mg/g for 80 °C and 95 °C prepared IONPs, respectively, going until ~60 mg/g for the corresponding SiO_2_-shell functionalized nanoparticles. Quite different photocatalytic and sorption behavior (under UV and dark) of the two sets of iron oxide nanomaterials obtained at 80 and 95 °C, supported by the SEM images, XRD, and BET information, became closer for most all the functionalized samples, with an efficiency of over 95%, during the first experimental cycle.

PFO and PSO kinetics are suitable to describe the degradation process under M1 series and M2 series, respectively. M1 sample shows faster degradation activity than M2 and under the corresponding SiO_2_-shell functionalized photocatalysts the MB degradation is three–four times faster. The particle sizes varying from 26–33 to 16–24 nm for the iron oxide samples (M1 and M2) show prevention of particle agglomeration in functionalized samples, changing particle sizes from 16–29 to 12–30 nm in the case of SiO_2_ and SiO_2_/ZnO core-shell samples, M(1/2)-S(1/2), respectively. Steep increase of the specific surface (5–6 times), i.e., increase of the number of active adsorption/degradation centers, causes enhancement of the photocatalytic activity by compensating the structural effects generated by differences in precipitation temperature. ZnO doping of the SiO_2_ shell enables slight enhancement of the photocatalytic activity under UV exposure. Significant differences in their efficiency occur after three operating cycles.

The tested thin films (500 ± 100 nm) show photocatalytic activity that increases by SiO_2_ surface functionalization which is more consistent for the films with ZnO-doped silica shell. Thus, the adsorption capacity increased by 1–1.5 mg/g for samples M(1/2)-S2 with respect to M(1/2)-S1 samples. A future work aims at structural, morphological, magnetic, and photocatalytic characterization of the IONPs containing membranes.

## Figures and Tables

**Table 1:** Structural and physical parameters of investigated MNPs.

Sample	XRD Results/Structural Parameters						TexturalParameters		Magnetic Parameters	
	Phases	wt%	2θ (°) (311)	FWHM (°)	Lattice Parameter a = b = c (Å)	Cryst Size (Å)	S_BET_ (m^2^g^−1^)	Pore Volume (cm^3^g^−1^)	Ms [emu/g]	Quality Factor
M1	Fe_3_O_4_	78	35.431	0.391	8.409(6)	22(1)	63.3	0.393	70.12	4.6 × 10^−5^
	γ-Fe_2_O_3_	22	—	—	—	—				
M1-S1	Fe_3_O_4_	33	35.426	0.301	8.413(4)	29(2)	334.7	0.498	34.39	5.7 × 10^−4^
	γ-Fe_2_O_3_	67	—	—	—	—				
M1-S2	Fe_3_O_4_	24.3	35.283	0.374	8.414(3)	23(1)	338.0	0.473	31.41	4.5 × 10^−5^
	γ-Fe_2_O_3_	75.7	—	—	—	—				
M2	Fe_3_O_4_	37.08	35.363	0.504	8.4107(9)	17(2)	70.6	0.322	66.82	6.0 × 10^−4^
	γ-Fe_2_O_3_	61.16								
	Fe(HO)_2_	1.76								
	Fe_3_O_4_	11.32			8.3517(7)					
M2-S1	γ-Fe_2_O_3_	70.84	35.595	0.487		18(2)	364.8	0.387	19.5	9.5 × 10^−4^
	Fe(HO)_2_	17.84								
M2-S2	Fe_3_O_4_	15.28	35.582	0.478	8.3554(4)	18(2)	396.7	0.391	24.02	7.5 × 10^−4^
	γ-Fe_2_O_3_	72.55								
	Fe(HO)_2_	12.17								
Reference (JCPDS no. 01-076-1849)			35.41	—	8.400	—				

**Table 2:** Kinetic parameters of first stage of MB adsorption onto M(1/2)-S(1/2) (linear fit up to 25 min).

Sample	M1	M1-S1	M1-S2	M2	M2-S1	M2-S2
Intraparticle diffusion (ID) q_t_ = k_ID_ t^0.5^						
k_id_ (mg/g h)	7.46676 ± 0.22619	14.3152 ± 1.55213	14.46265 ± 1.54519	3.22096 ± 0.27047	11.91919 ± 0.91285	13.92478 ± 1.11899
R^2^	0.99543	0.94448	0.94601	0.96594	0.97151	0.96872

**Table 3:** Kinetical parameters for the incipient photocatalysis process of MB degradation under UV irradiation (linear fit bellow 30 min).

Sample	k_1_ (min^−1^)	R^2^	k_2_ (mg dm^−3^ min^−1^)	R^2^
M1	0.04861 ± 8.9805 × 10^−4^	0.9989	0.03945 ± 0.00146	0.99323
M1-S1	0.20105 ± 0.02947	0.9939	0.40462 ± 0.03925	0.95508
M1-S2	0.23024 ± 0.045	0.8971	0.5188 ± 0.07531	0.90468
M2	0.01203 ± 0.00136	0.9398	0.0086 ± 8.9857 × 10^−4^	0.94820
M2-S1	0.12413 ± 0.0148	0.9336	0.31748 ± 0.0163	0.98690
M2-S2	0.13549 ± 0.01641	0.9314	0.39957 ± 0.00884	0.99707
*Equation [9]*	*ln(C_t_/C_0_) = k_1_t*		*(1/C_t_−1/C_0_) = k_2_t*	

## Data Availability

All the data supporting the findings of this study are available within the article.

## Supplementary Materials

The following supporting information can be downloaded at: https://www.mdpi.com/article/10.3390/membranes12050466/s1, Figure S1: Absorbance spectra of the MB solution, before and after 120 min exposure to UV (a) and dark (b) in the presence of investigated samples; Figure S2: Digital picture of MB recovered from supernatant–water-investigated samples (photocatalytic test 120 min. exposure to UV (a) and dark (b)); Figure S3: Absorbance spectra of the MB recovered solution from supernatante-water-M1/M2 and corresponding to the core-shell (M1-S1/2 and M2-S1/2) nanopowders exposed to UV (a) or dark (b).
