# Supplementary material for: Magnetic Core-Shell Iron Oxides-Based Nanophotocatalysts and Nanoadsorbents for Multifunctional Thin Films
Source: Membranes (Basel). 2022 Apr 26;12(5):466. doi: 10.3390/membranes12050466 (PMC9144956; doi:10.3390/membranes12050466)
Supplement: Supplementary file 1 [file membranes-12-00466-s001.zip › membranes-1639840-supplementary.pdf]

membranes

MDPI

Type of the Paper (Article)

SUPPLEMENTARY INFORMATION

Magnetic Core-Shell Iron Oxides-based Nanophotocatalysts and Nanoadsorbents for Multifunctional Thin Films and Membranes

Viorica MUȘAT[1*], Nicolae STĂNICĂ[2], Elena Maria ANGHEL[2*], Irina ATKINSON[2], Dana CULIȚĂ[2], Silviu POLOŞAN[3], Lenuța CRINTEA (CĂPĂȚÂNĂ)[1], Alina CANTARAGIU CIOROMILĂ[4], Cristian-Teodor BURUIANĂ[5] and **Oana CARP**[2]

**Corresponding authors: viorica.musat@ugal.ro, manghel@icf.ro*

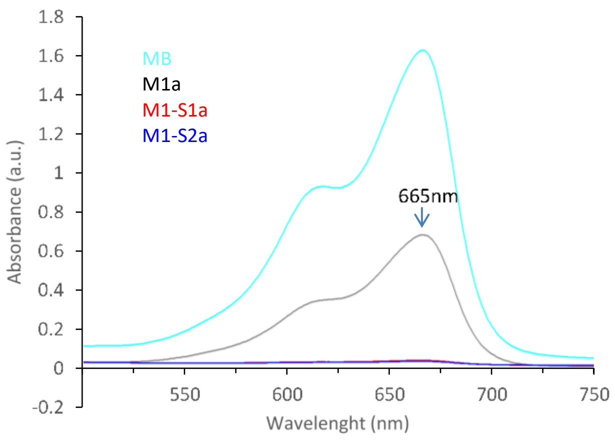

[1]Corresponding authors. *E-mail address*: viorica.musat@ugal.ro (Viorica Musat), manghel@icf.ro (Elena Maria Anghel)

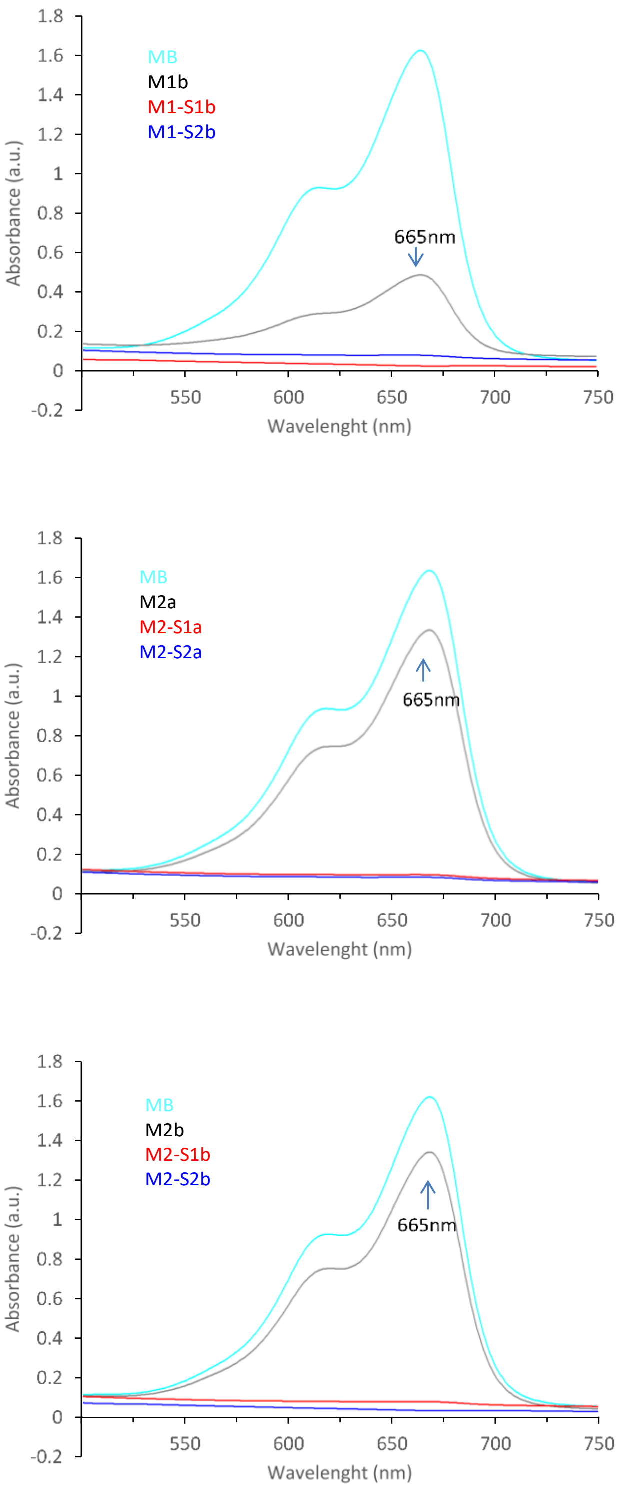

Figure S1: Absorbance spectra of the MB solution, before and after 120 min exposure to UV (a) and dark (b) in the presence of investigated samples

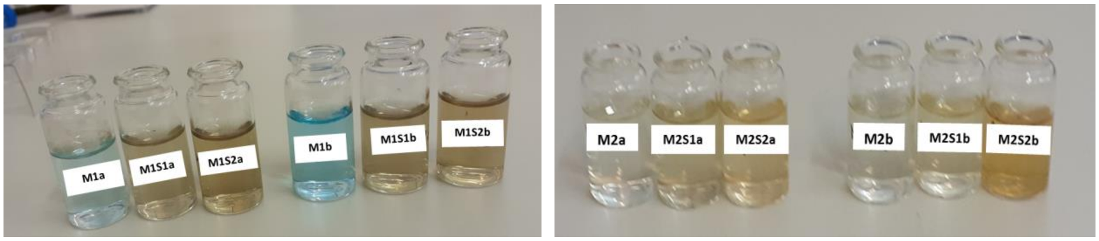

Figure S2: Digital picture of MB recovered from supernatant-water-investigated samples (photocatalytic test 120 min. exposure to UV (a) and dark (b))

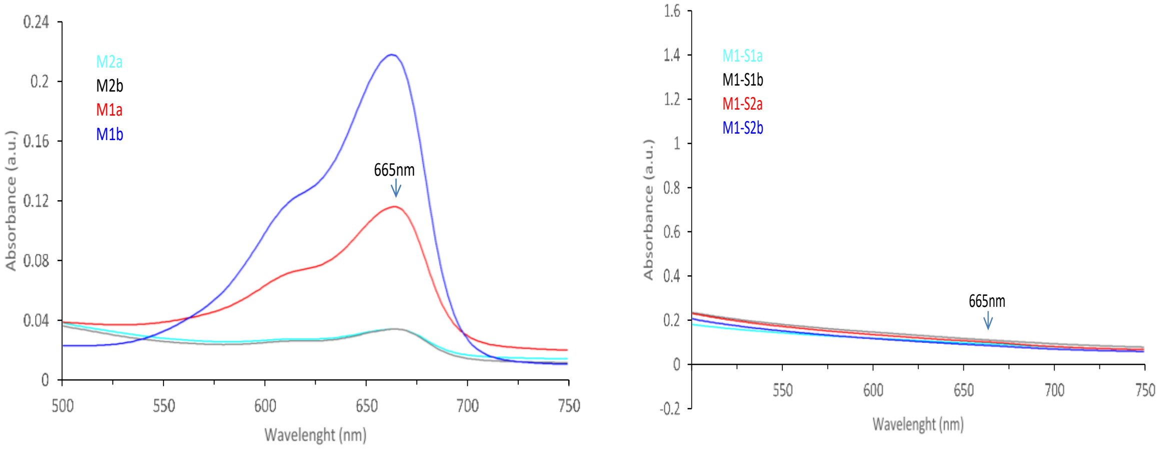

Figure S3: Absorbance spectra of the *MB recovered* solution from supernatante-water-M1/ M2 and corresponding to the core-shell (M1-S1/2 and M2-S1/2) nanopowders exposed to UV (a) or dark (b)
